# Near-Random Distribution of Chromosome-Derived Circular DNA in the Condensed Genome of Pigeons and the Larger, More Repeat-Rich Human Genome

**DOI:** 10.1093/gbe/evz281

**Published:** 2019-12-27

**Authors:** Henrik Devitt Møller, Jazmín Ramos-Madrigal, Iñigo Prada-Luengo, M Thomas P Gilbert, Birgitte Regenberg

**Affiliations:** 1 Department of Biology, University of Copenhagen, Denmark; 2 Department of Biology, Institute of Biochemistry, ETH Zürich, Switzerland; 3 The GLOBE Institute, University of Copenhagen, Denmark; 4 NTNU University Museum, Trondheim, Norway

**Keywords:** circular DNA, repetitive elements, genome size, evolution, genome condensation, mutation rate

## Abstract

Extrachromosomal circular DNA (eccDNA) elements of chromosomal origin are known to be common in a number of eukaryotic species. However, it remains to be addressed whether genomic features such as genome size, the load of repetitive elements within a genome, and/or animal physiology affect the number of eccDNAs. Here, we investigate the distribution and numbers of eccDNAs in a condensed and less repeat-rich genome compared with the human genome, using *Columba livia domestica* (domestic rock pigeon) as a model organism. By sequencing eccDNA in blood and breast muscle from three pigeon breeds at various ages and with different flight behavior, we characterize 30,000 unique eccDNAs. We identify genomic regions that are likely hotspots for DNA circularization in breast muscle, including genes involved in muscle development. We find that although eccDNA counts do not correlate with the biological age in pigeons, the number of unique eccDNAs in a nonflying breed (king pigeons) is significantly higher (9-fold) than homing pigeons. Furthermore, a comparison between eccDNA from skeletal muscle in pigeons and humans reveals ∼9-10 times more unique eccDNAs per human nucleus. The fraction of eccDNA sequences, derived from repetitive elements, exist in proportions to genome content, that is, human 72.4% (expected 52.5%) and pigeon 8.7% (expected 5.5%).

Overall, our results support that eccDNAs are common in pigeons, that the amount of unique eccDNA types per nucleus can differ between species as well as subspecies, and suggest that eccDNAs from repeats are found in proportions relative to the content of repetitive elements in a genome.

## Introduction

It has been shown that eukaryotic nuclear DNA can excise from chromosomes and form circular molecules that are retained within the cell. These extrachromosomal circular DNA (eccDNA) elements can, in certain cases, replicate and propagate in cells as semistable genetic elements (Kanda et al. 2001; Gresham et al. 2010; Vogt et al. 2014; Meng et al. 2015; Shoura et al. 2017; Turner et al. 2017) and have so far been found in all eukaryotic species studied to date (Gaubatz 1990; Paulsen et al. 2018 for reviews). Whole-genome sequence analyses of eccDNA from yeast, nematodes, ciliates, and mammalian species report eccDNA in sizes from <100 bases up to several hundred thousand base pairs, carrying not only complete or partial genes but also intergenic sequences (Møller, Parsons, et al. 2015; Møller et al. 2016; Kumar et al. 2017; Shoura et al. 2017; Møller, Mohiyuddin, et al. 2018; Yerlici et al. 2019). EccDNAs are likely players in genome evolution because they are found as intermediates of deletions (Gresham et al. 2010) and eccDNAs can serve as templates for reinsertion into chromosomes in systems as diverse as cows (Durkin et al. 2012), yeast (Galeote et al. 2011; Demeke et al. 2015), and human cancer cells (Vogt et al. 2014; Turner et al. 2017).

A number of repetitive regions and genes in tandem arrays have been found to form eccDNA in yeast, including ribosomal RNA genes (rDNA), histone genes, and genes encoding plasma-membrane transporters (Sinclair and Guarente 1997; Libuda and Winston 2006; Gresham et al. 2010; Møller, Parsons, et al. 2015; Møller et al. 2016). Also DNA from animals and plants is known to form eccDNA from tandem gene arrays and repetitive regions, such as telomeres, rDNA, and retrotransposons (Gaubatz and Flores 1990; Cesare and Griffith 2004; Cohen et al. 2007; Navrátilová et al. 2008; Møller, Larsen, et al. 2015; Lanciano et al. 2017; Møller, Mohiyuddin, et al. 2018).

However, it is largely unknown whether structural features such as the fraction of repetitive elements within a genome and whether biotic factors affect eccDNA presence. Birds represent an appealing model to explore these questions. The avian genome contracted during its evolution, to a current size of around 1 Gb (Zhang, Li C, et al. 2014). This contraction involved reduction of intron and noncoding regions to circa half size of that in mammals and around one-third of reptilian genomes (Zhang, Li C, et al. 2014). Compared with the genomes of some of the closest ancestors of birds, the American alligator, and green turtle, repetitive elements in the form of short interspaced nuclear elements (SINEs) have been reduced 5–14-fold in the bird genome of the rock pigeon (*Columbalivia*), whereas DNA transposons and long interspaced nuclear elements (LINEs) have been reduced 2–3-fold and 0.5-fold, respectively (Zhang, Li C, et al. 2014). It has been argued that this genomic condensation may have been associated with the higher metabolic cost of powered flight, because the genomes of flying birds are more condensed than those of flightless birds (Wright et al. 2014; Kapusta et al. 2017). This trend is also found in flying bats, a nonavian lineage, where genome sizes are similarly more condensed compared with related mammals (Kapusta et al. 2017; Teeling et al. 2017). Yet, the consequences for birds having a higher metabolic rate and a smaller genome with less repetitive elements in respect to structural somatic mutations have not been explored.

We hypothesized that 1) chromosome-derived eccDNA from repeats is proportional to the number of repetitive elements within a genome, due to the near-random effect of DNA damage and 2) birds with different lifestyles and metabolism would contain dissimilar eccDNA counts due to varying oxidative stress levels. To assess this, we determined the types and genomic origin of eccDNA in the domestic rock pigeon, *Columba livia domestica*. We leveraged the low genetic diversity present in pigeon breeds (Shapiro et al. 2013) to evaluate the effect of flight activity, age, and breed in respect to the production of eccDNA, without the potential confounding effect of genetic variability encountered if comparing different species. Using the Circle-Seq method (Møller, Mohiyuddin, et al. 2018), we purified, sequenced, and mapped eccDNA from blood and breast muscle of three pigeon breeds with different flight activity. This analysis also allowed us to access DNA circularization events from repetitive and unique elements in the pigeon genome and compare these to DNA circularization events in the human genome from skeletal muscle tissue. The data support our hypothesis that eccDNA from repeat elements is proportional to the level of repeats in a genome. On the other hand, and contrary to our hypotheses, the biotic factors tested had little to no correlation with eccDNA presence.

## Materials and Methods

### Samples Description

#### Pigeon Collection

A variety of domestic rock pigeon (*C.livia domestica*) breeds were obtained including flying homing pigeon (HP, *n* = 15 from two different lofts) and nonflying king (K, *n* = 10 from two different lofts) and Danish Suabian (S, *n* = 2 from a fifth loft). Age, gender, and breed type were annotated for each bird. For homing pigeons, the total traveled flight distances in competitions during lifespans were annotated. Note that homing pigeons also flew additionally 500–1000 km/year as part of training exercises. In agreement with local pigeon breeders, each bird was sacrificed by rapid rotation of the body causing instant neck breakage. Birds were photographed and wingspans (cm), hearts (g), breasts (g), and body weight (g) were measured. Breast tissues were rinsed with ethanol and sliced with a sterile scalpel in cubes of ∼125 mm^3^ and submerged in 1.75-ml RNAlater (Qiagen) for 24 h at 4 °C. RNAlater was removed and muscles were stored at −80 °C until further use. For blood collection, heads were pulled off to collect 5–17.5-ml blood by gravity flow from the neck into a 50-ml tube. The blood was placed at −20 °C until further use. Each sample was denoted according to the bird age and type; that is, homing pigeon (HP), king (K), and Danish Suabian (S). For example: 2-HP9B, meaning 2-year-old homing pigeon with ID number 9, replicate B.

#### Circle-Seq Methodology

 

#### Blood Samples

Cells were thawed at room temperature, 1× volume phosphate-buffered saline (PBS) was added to each sample, and tubes were centrifuged at 3,500 × g for 15 min. Cells from the tube bottom were collected (0.5 ml) and a 1:10 PBS diluted cell solution was used for nuclei counting with Solution 17 according to protocol (NucleoCounter NC-3000, Chemometec). Cell concentrations were adjusted to 3.3E + 06 cells/aliquot and cells were lysed as previous described (Møller, Mohiyuddin, et al. 2018) and pellets were stored at −80 °C until further use.

#### Muscle Samples

Aliquots of 4-mg air-dried breast muscle tissue (see supplementary fig. S1, Supplementary Material online) were prepared as described (Møller, Mohiyuddin, et al. 2018) and then stored at −80 °C until eccDNA purification.

#### eccDNA Purification

EccDNAs from somatic tissues of pigeons were purified by Circle-Seq (Møller, Mohiyuddin, et al. 2018), using 4-mg tissue or ∼1–2 million blood cells as entry material. Soma cells were suspended in 0.615-ml L1 solution (Plasmid Mini AX; A&A Biotechnology) and lysed by adding 15-µl Proteinase K (>0.1 U/µl, Life Technologies) followed by overnight incubation at 50 °C with agitation at 700 rpm (Eppendorf Thermomixer). For complete lysis of muscle, additional 15-µl Proteinase K was added next day and extra 24-h incubation was completed at 50 °C with agitation at 700 rpm. After cell lysis, each sample was split in a 1:1 ratio. The first sample was used to assess the input DNA concentration by quantitative PCR after completed protocol for genomic DNA purification (AX mini tissue or blood kit; A&A Biotechnology) and the second sample was used for eccDNA detection by continuing the Circle-Seq method. In brief, samples for eccDNA purification were added extra 300-µl L1 solution. DNA in each cell lysate was then alkaline treated for rapid denaturing–renaturing, followed by column chromatography on an ion-exchange membrane column to separate chromosomal DNA, lipids, and protein from eccDNAs according to protocol (Plasmid Mini AX; A&A Biotechnology). To enhance DNA precipitation after column elution, samples were incubated at −20 °C for 50–55 min, followed by extended centrifugation at 9,788 × g for 30 min at 2 °C and subsequent ethanol wash of pellets according to protocol. Precipitated and air-dried DNA was dissolved in 50-µl ultraclean sterile water, measuring total DNA of 20–359 ng per tissue sample (*n* = 31) and 73–228 ng per blood sample (*n* = 8) by Qubit dsDNA High Sensitivity assay.

#### Linear and Mitochondrial DNA Removal

Remaining linear DNA was removed by exonuclease, initially assisted by the rare-cutting endonuclease *Pac*I that beside chromosomes also cleaved circular mitochondrial DNA (mtDNA). DNA was treated with 2 FastDigest Units *Pac*I (TTAA^TAA) (Thermo Scientific) and incubated at 37 °C for 16 h. *Pac*I was thermally inactivated at 65 °C for 20 min, cooled down on ice for 10 min and then retreated with 2 FastDigest Units *Pac*I at 37 °C for 8 h. After thermal inactivation of *Pac*I, linear specific exonuclease treatment was set up at 37 °C. For this, chromosomal DNA digestion was carried out continuously for 1 week (165 h), adding additional ATP and DNase every 24 h (30 units per day, total 180 units) according to protocol (Plasmid-Safe ATP-dependent DNase, Epicentre). Finally, exonuclease was heat inactivated at 70 °C for 30 min.

#### Rolling-Circle Amplification of eccDNA

Approximately 5% (10 µl) of the total volume of each eccDNA-enriched sample was used as template for phi29 polymerase reaction (REPLI-g Midi Kit) after adding two internal plasmid controls (pBR322 and pUG72, 10,000 copies/plasmid) to all samples. The DNA was amplified at 30 °C for 2 days (48–49 h).

#### Plasmids

pBR322 (4,361 bp; New England Biolabs) and pUG72 (3,988 bp; originally pJJH726, EUROSCARF) were maintained in *Escherichia coli* and purified with a plasmid miniprep kit (GeneJet, Thermo Scientific). Each of the plasmids pBR322 and pUG72 was added post exonuclease treatment in ∼10,000 copies to each sample, 4.7E-05 ng and 4.3E-05 ng, respectively.

#### Sequencing of eccDNA from Tissue and Blood

Phi29-amplified DNA was sheared by sonication to mean fragment size 300 nucleotides (Covaris LE220). DNA was purified (Ampure bead) and 300-ng fragmented DNA per sample was loaded onto a robotic Apollo 324 system (IntegenX) for library preparation (Wafergen’s PrepX ILM DNA Library Kit), adding adapters and barcode index labels (hexamer oligos). Samples were multiplexed and sequenced as 2× 75-nucleotide paired-end reads (Illumina HiSeq 2000 Rapid flowcell), obtaining 34.2–66.4 million paired-end reads/sample.

### Mapping Pipeline for Circle-Seq Data

#### Reads Processing and Mapping

Paired-end sequencing reads were demultiplexed and only reads with a perfect match with the corresponding six nucleotides index were kept. Sequencing adapters, low quality stretches (runs with quality of 2), and leading and tailing N’s were trimmed from the raw reads using AdapterRemoval 2.0 (Schubert et al. 2016). Reads shorter than 30 bp and singletons were discarded. Bowtie2 (Langmead and Salzberg 2012) was used to map the reads to a custom reference genome composed by the *C.livia* genome assembly (version 1.0) (Shapiro et al. 2013), the reference mitochondrion sequence (GU908131) (Kan et al. 2010), and plasmid sequences. Mapping was performed by Bowtie2, using –local option, in order to detect partially mapped reads at potential DNA break points.

#### Identification of eccDNA

**Fig. 1.**
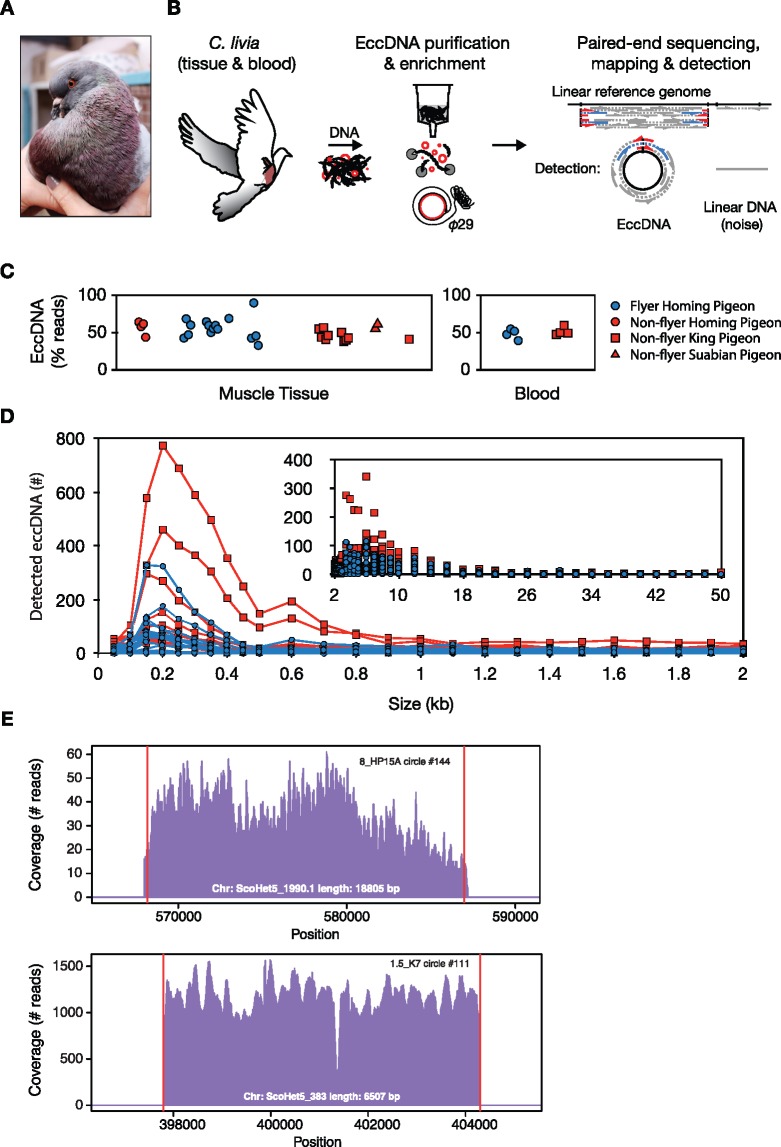
—EccDNA purification, detection, and size distribution in pigeon muscles and blood. (*A*) Picture of a king pigeon (*Colombia livia*). (*B*) Schematic description of the Circle-Seq method used for genome-wide profiling of eccDNA from pigeon, that is, eccDNA elution through a column ion-exchange membrane, removal of reminiscent linear DNA by exonuclease, rolling-circle amplification by phi29 polymerase, and finally paired-end sequencing, mapping, and detection of eccDNA from paired-end reads. EccDNAs were recorded with high confidence when at least two reads supported the circle junction (discordant reads, blue; soft-clipped or split reads, red) and read coverage was more than 80% from concordant read pairs, gray. (*C*) Read percentage on detected eccDNA relative to total mapped reads to the pigeon genome. *x* axis, sample separation by group and age in arbitrary units (not shown). (*D*) Length distribution of eccDNAs in muscle samples, each from ∼1E + 06 muscle nuclei (*n* = 27), grouped in bins of 50 bp (0–500 bp), 100 bp (500–3000 bp), 1,000 bp (3,000–50,000) intervals. (*E*) Representative read-coverage plots from a locus in homing pigeon (8_HP15A, upper plot) and a locus in king pigeon (1.5_K7, lower plot), detected as original sites of eccDNA formation.

In order to identify eccDNAs in the sequencing data, we followed two independent approaches: first, by identifying discordantly mapped paired-end reads, and second, by using Socrates (Schröder et al. 2014) to identify reads that could support rearrangements that could match circular DNA structures. The first approach relied on the orientation of mapped paired-end reads because reads spanning the breakpoint in a circular element would map discordantly to the reference genome (see fig. 1*b*, blue reads). In detail, each identified discordant paired-end read pair fulfilled the following criteria:

1. Paired-end read pairs mapped discordantly, that is, the reads map in opposite orientations and had a negative insert size (distance between the 5′-most positions of each read in a given pair).
2. Both reads in the discordant read pair could be mapped uniquely and to the same scaffold.
3. Each read in the discordant read pair had a minimum mapping quality of 10.
4. Settings of overlapping read pairs had a negative insert size of at least the read length plus five nucleotides (minimum five nonoverlapping bases).

Discordant paired-end reads were used to define potential eccDNA-derived regions and overlapping regions were merged to create an eccDNA list for each sample.

As a second approach, we used Socrates (Schröder et al. 2014) to identify junctions potentially formed after DNA circularization. Socrates was run on bowtie2 aligned reads to identify regions derived from potential rearrangements, using default parameters. The results from both approaches were merged, creating a combined list of putative chromosomal-derived eccDNA coordinates. To assess the support of those regions, we estimated the internal read coverage, depth of coverage, and amount of discordant read pairs supporting each region. Finally, we created a list of high confidence eccDNA defined for those fulfilling listed criteria: 1) the region had at least 80% read coverage, 2) the eccDNA structure was identified by discordant and partially mapped reads (two independent approaches) or it was supported by at least two discordant pairs, and 3) it could be anchored to a unique region in the genome (minimum 10% of region could be mapped uniquely). All analyses presented here were done using the high confidence data set.

### Additional Data Analyses

 

### Quantification of Genomes per Sample

The number of genomes per sample was quantified by quantitative PCR at the *C.livia* ephrin-B2 gene (*EFNB2*). Genomic DNA preparations, completed after proteinase K digestion, were used as templates to estimate input DNA by qPCR and likewise, 1-week exonuclease-treated DNA samples were used as template to estimate remaining linear DNA (*EFNB2* copies) as well as mtDNA. Oligos for *EFNB2* were 5′-GACCAGGGTTGATTTGCCTAA-3′ and 5′-TACCAGCCGTGTGAACTCTG-3′ (product 119 bp) and for mtDNA 5′-TGCTAAGACTCGCAGGACATT-3′ and 5′-CTGTTGGTTTAGGCGTCTGG-3′ (product 136 bp). All reactions were run in quadruplicates in a Quant Studio 7 Flex qPCR machine (Applied Biosystems) in 10-µl reactions with 2-µl template (exonuclease-treated samples), 60 nM primers, and 5 µl SYBR Green PCR Master Mix (Applied Biosystems). Reaction conditions were 10 min at 95 °C followed by 40 cycles of 15 s at 95 °C and 60 s at 60 °C. To verify reaction specificity, melting curves were generated and lengths of PCR products were verified by conventional agarose gel electrophoresis. Purified human genomic DNA in eight serial dilutions was used at each run to produce a standard curve. Genome counts were calculated, assuming two copies of *EFNB2* per genome, a molar mass per base pair of 650 g/mol, a genome length of 1.1 × 10^9^ bp and 2.257E-03 ng DNA/diploid cell. Two to four independent qPCR experiments were conducted for each experiment reporting genome counts as average between all experiments. Concentrations of standards were measured using Qubit dsDNA High Sensitivity assay (Life Technologies).

### Saturation Plots

To assess the effect of sequencing depth in the detection of eccDNA elements, we randomly sampled 0.25, 0.5, 1, 2.5, 5, 7.5, 10, 12.5, and 15 million mapped reads from each sample and estimated the eccDNA counts to generate decimation plots (supplementary fig. S6, Supplementary Material online).

### Annotation of eccDNA Elements

We used the annotation of the pigeon genome version BGI_colLiv_0.0. Overlaps between detected eccDNA coordinates and annotated genes in the pigeon genome were categorized into four groups: 1) eccDNA contained a gene, 2) eccDNA contained a complete gene or many genes, 3) eccDNA partially overlapped (10% reciprocal overlap) with annotated gene(s), and 4) eccDNA derived from an intergenic region.

### Enrichment Analyses of eccDNA Content

To evaluate if eccDNAs were enriched in particular regions of the genome, we compared the location of the detected eccDNA with a set of randomly selected regions of the genome. First, we created a list of nonredundant circular regions by pooling detected eccDNAs from all pigeon samples and merging overlapping regions (*n* = 29.327 eccDNAs), using bedtools (merge -d 0). Then, we constructed a random data set by randomly sampling regions with the same size distribution as the original data set. We repeated the procedure ten times, so that we had ten random independent data sets to compare with. For each of them, we measured the overlap of both data sets with annotated features in the genome: genes, 5′-untranslated region (5′-UTR) and 3′-UTR, repetitive elements, and exons, requiring, for each case, a minimum 10-bp overlap. We assessed the statistical significance in this comparison by preforming a chi-square test comparing the proportion of each genomic feature in each of the ten random data sets with the proportions observed in the real data (supplementary table S3, Supplementary Material online). For the comparison of the distribution of GC content in detected eccDNAs with randomly selected regions (supplementary table S3, Supplementary Material online), we used a Student’s *t*-test (as implemented in *R*).

### BlastN and Taxonomy

To explore the unmapped fraction of reads, we randomly sampled a subset of reads (10,000 reads, only first read in sequenced pairs). Then, we performed a search for sequence similarity, using the BlastN algorithm and the NCBI nucleotide database *nt*, allowing up to ten best-scoring hits (Altschul et al. 1997), followed by a taxonomy assignment at the phylum level using MEGAN (Huson et al. 2007).

For instance, in many of the muscle samples, we also detected scattered read fractions from bacterial DNA, primarily consisting of benign, Gram-positive *Rhodococcus erythropolis* (stain CCM2595, 6,281 kb), previously found in the microbiota of wild birds and on egg-shells (van Dongen et al. 2013; Grizard et al. 2015) and in two muscles and in all blood samples, a Torque teno virus was identified that has been found in many different vertebrates, having some similarity to chicken anemia virus (Hino and Miyata 2007) (supplementary fig. S3, Supplementary Material online).

### eccDNA Normalization to Plasmids

We spiked-in pBR322 and pUG72 plasmids, 10,000 copies each, to all purified eccDNA samples just before rolling-circle amplification by phi29 polymerase. A known plasmid number in each sample, allowed us to compare samples, as the extent of plasmid amplification (% mapped reads to plasmids) would indirectly infer how many eccDNAs were present. This is based on the expectation that the phi29 polymerase (Qiagen), primed by random hexamer oligo’s, is unbiased and will initiate amplification of all available DNA templates equally. If so, abundant amounts of eccDNAs will lead to less amplification of plasmids and vice versa. Thus, for each sample, we calculated a plasmid map percentage for normalization; % reads/plasmid = (total number of mapped reads on plasmids × 100)/total number of all mapped reads. The numbers were then compared, calculating relative numbers of eccDNAs per sample: relative number of eccDNAs/sample = (number of detected eccDNAs/samples)/(% reads/plasmid).

### Repetitive DNA Quantification

The Circle-Seq human data from a recent study (Møller, Mohiyuddin, et al. 2018) were downloaded from the Sequence Read Archive (SRA BioProject ID: PRJNA419440). The reads were aligned using bowtie2 (v2.2.4) with default parameters to a custom reference containing the human genome excluding the mitochondria (hg38 assembly) and the set of plasmids used in the Circle-Seq procedure (p4339, pSH63, pUC19_yEGFP, pUG72, pBR322, YGPM25009_18kb, and YGPM3k20_chrV_26_kb). The resulting sam files were bam converted, sorted, and indexed using Samtools (Li et al. 2009) (v1.5). Repeats were then quantified by counting the number of reads aligned to the annotated repeat classes in the human hg38 genome,—Dec 2013—RepeatMasker open-4.0.5 - Repeat Library 20140131 (source: A.F.A. Smit, R. Hubley, and P. Green, RepeatMasker at http://repeatmasker.org; last accession January 4, 2020) using BedTools multicov (Quinlan and Hall 2010) (v2.26.0-148-gd1953b6). The number of reads aligned to every repeat class was counted using BedTools groupby. In order to make every sample comparable, a series of normalizing steps were applied. First, all the samples were normalized by library size dividing the reads aligned to every repeat class by the total number of reads aligned to the nuclear genome. Second, and in order to allow for the comparison between species with different repeat content, the reads aligned to every repeat class were divided by the percentage of repetitive bases for a given repeat in the nuclear genome, which was computed from the repeat masked file for every genome.

For the pigeon data, we estimated the overlap between the annotated repeats (source: RepeatMasker of the pigeon genome [Shapiro et al. 2013]) and mapped reads, using *bedTools multicov* at default parameters.

## Statistical Analyses

For all data where a Gaussian distribution could be assumed, average and standard deviations (SDs) were calculated. Median values were used to describe data relating to size, number, and content of eccDNA, as the underlying data distribution was unknown.

Mann–Whitney *P* values were computed in R or Prism. Obtained *P* values were considered significant if *P* < 0.05 (*), *P* < 0.01 (**), *P* < 0.001 (***), and *P* < 0.0001 (****). Monte Carlo simulations and statistical significance enrichment analyses of eccDNA contents were done by a Pearson chi-square test, using R (v3.4.2). EccDNA enrichment analysis of genomic features was considered significant for *P* < 0.05. Statistical power of Mann–Whitney comparisons between different pigeon breeds was done by post hoc power analyses. For every paired comparison, we assumed that the underlying distribution for every group was a normal distribution (although not defined), parameterized by the sample mean and variance. We performed 10,000 sampling rounds from the pair of distributions, using the sample size in each group, followed by Mann–Whitney *U* test between the sampled values with the significance level *P* < 0.05. A Wilcoxon test (as implemented in R) was used to test the differences between the repeat proportions in the human samples against the pigeon samples.

## Results

### Characterization of eccDNA from Pigeons

We characterized the genomic distribution and relative abundance of eccDNAs in blood cells and breast muscle, termed flight muscle (Bishop and Butler 2015), of domestic rock pigeons (*C. livia domestica*) (fig. 1*a*), using the Circle-Seq method (fig. 1*b*) (Møller, Mohiyuddin, et al. 2018). DNA was collected from pigeons of different age: 6 weeks up to 8 years, and different breeds: the highly active homing pigeon (*n* = 11, flying; *n* = 4, preflying), the flightless king pigeon (*n* = 10, caged), and the Danish Suabian breed with poor flight ability (*n* = 2, caged) (supplementary table S1, Supplementary Material online). Circular DNA was purified from ∼1 million nuclei per sample (supplementary fig. S1, Supplementary Material online). The eccDNA was enriched by enzymatic removal of linear DNA (supplementary fig. S2, Supplementary Material online) followed by rolling-circle amplification and paired-end sequencing. Paired-end reads from sequenced eccDNA-libraries were mapped to the rock pigeon reference genome (Shapiro et al. 2013). In order to identify eccDNA in the sequencing data, we identified reads that mapped at or near DNA breakpoints, that is, putative excision points in the genome. High confidence eccDNAs were recorded based on following criteria: 1) a minimum of two sequencing reads identified at the circular DNA junctions (discordant and soft-clipped reads), 2) the locus had more than 80% read coverage, and 3) at least 10% of the region was covered by reads with high mapping quality (∼unique sequence). With this conservative cutoff, around 50% (54.1% tissue; 49.0% blood, median) of all reads mapping on the pigeon genome were recorded as eccDNAs (fig. 1*c*).

Overall, we detected similar median counts of eccDNA in blood (992 per 1.1E + 06 nuclei, *n* = 8) and skeletal muscle (943 per 5.5E + 05 nuclei, *n* = 27), although the range was considerably broader for muscle (24–5,744, *n* = 27) than for blood (786–1,671, *n* = 8) (supplementary table S2, Supplementary Material online). Despite individual variation in eccDNA counts between pigeons, eccDNAs followed a similar length distribution, ranging from below 100 bases up to detected breakpoints 48.1 kb apart (fig. 1*d*). The majority of detected eccDNAs were around ∼200 bp, consistent with previous reports on eccDNA from humans and mice (Shibata et al. 2012; Kumar et al. 2017; Zhu et al. 2017; Møller, Mohiyuddin, et al. 2018; Paulsen et al. 2019). We have previously recorded eccDNA from human skeletal muscle, *vastus lateralis*, with identical methods and a similar number of nuclei. Pigeons have less eccDNA types (9–10-fold) in muscle nuclei (*n* = 27) in comparison to eccDNA counts in muscle nuclei from humans, having a three times larger genome (median ∼9,000 per 1E + 06 nuclei, *n* = 16) (Møller, Mohiyuddin, et al. 2018).

We validated the efficacy of our method using several approaches. First, we confirmed detection of internal controls from circular mtDNA (17.2 kb) and from two spike-in plasmids (∼4 kb, 20.000/sample) in all samples investigated (supplementary fig. S3, Supplementary Material online). Second, we used our bioinformatics pipeline to search for eccDNAs in previously published whole-genome sequencing data of pigeon (Shapiro et al. 2013) to get an estimate of the number of eccDNAs in non-eccDNA-enriched data. By this approach, we found that the background from linear noise (or eccDNA) corresponded to a maximum of 15% of the total detected eccDNAs (146 and 149 potential eccDNA structures in homing and king pigeon genomes, respectively). Third, the obtained read coverage across the pigeon genome also supported intensive read-signals from loci with eccDNAs relative to adjacent regions (fig. 1*e*). Besides this, we also found that linear DNA, in the form of *EFBN2* gene copies, had been reduced to <0.01% after plasmid-safe DNase treatment (fig. 1*b* and supplementary fig. S2, Supplementary Material online, copies in tissue 158 ± 267; in blood 145 ± 55). We were therefore confident that the majority of detected eccDNAs were true-positives. Finally, we discovered endogenous viral circular elements in the pigeon eccDNA data sets. This involved the circular circovirus (2.0 kb, single stranded), a common host within pigeons (Stenzel et al. 2014), detected with high abundance in homing pigeon 8-H14 (supplementary fig. S4*a*, Supplementary Material online), and the circular Torque teno virus (1.5–3.8 kb, single stranded), detected in two muscle samples and in blood from several birds (supplementary fig. S4*b*–*d*, Supplementary Material online).

### Pigeon eccDNAs Derived from Genic and Intergenic Regions

**Fig. 2.**
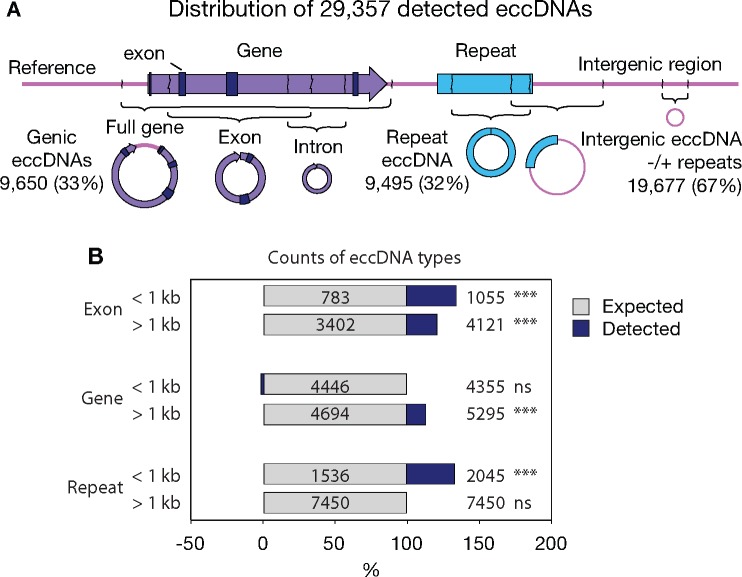
—Distribution of eccDNA across different genomic features. (*A*) Schematic representation of examined genomic features on eccDNA. Numbers correspond to total eccDNA counts from each feature and percent of total detected eccDNA counts across the 27 pigeon muscle samples, described in the legend to figure 1. EccDNAs overlapping with more than one genomic feature were categorized in several groups. (*B*) Barplots of observed counts of genomic features on eccDNAs (blue, *n* = 27) relative to expected counts (gray), grouped into small (<1 kb) and larger (>1 kb) eccDNA elements. Each bar represents the percentage of total and values show the absolute numbers. ***P < 0.001 (chi-square test).

To understand if certain genes or features were particularly prone to form circular DNA, we assessed the distribution of genomic features from the pigeon genome on eccDNA (fig. 2). A total of 29,357 unique eccDNAs were annotated in the 27 pigeon breast muscles, covering a combined length of 8.7% of the genome. EccDNAs derived from genic regions, intergenic regions, repetitive elements, or regions with several of these features (fig. 2*a*) supporting that the eccDNA distribution was not restricted to particular regions in the genome of pigeons.

In our data sets, a total of 1,083 complete genes (3.7%) were detected on eccDNAs (fig. 2*a*). Some of these gene-containing eccDNAs could potentially have physiological impact as studies have shown strong correlation between eccDNA presence and a selective growth and/or resistance advantage of cells, both in human tumor cell lines (Pauletti et al. 1990; Von Hoff et al. 1992; Kanda et al. 2001; Vogt et al. 2014; Meng et al. 2015; Turner et al. 2017), plant (Koo et al. 2018), and budding yeast (Libuda and Winston 2006; Gresham et al. 2010).

Small eccDNAs (<1 kb) have been reported to be enriched for genic and high GC content in the human and mouse genome, in particular 5′-UTR and exons (Shibata et al. 2012; Dillon et al. 2015). We compared the distribution of annotated sequence features on eccDNAs from 27 pigeons (*n* = 29,357) to 10 randomized version set of regions, drawn from the same number (*n* = 29,357) and size distribution. To account for potential differences based on eccDNA sizes, eccDNAs were divided into two categories (<1 kb and >1 kb). We found a significant enrichment of eccDNAs overlapping exons in both categories (1.4- and 1.2-fold, respectively). As well, 5′-UTRs and GC content were significantly overrepresented on eccDNAs (supplementary table S3, Supplementary Material online), in agreement with previous reports (Shibata et al. 2012; Dillon et al. 2015). In general, genic eccDNAs (>1 kb) and repeat eccDNAs (<1 kb) were significantly enriched (1.1- and 1.3-fold, respectively) from the expected by chance (fig. 2*b*). Overall, our result suggested that eccDNA carried different genetic elements in proportions that resemble the distribution of various genomic features.

### Distribution of Repeat-Classes in eccDNA Data Sets from Human and Pigeon Muscle

**Fig. 3.**
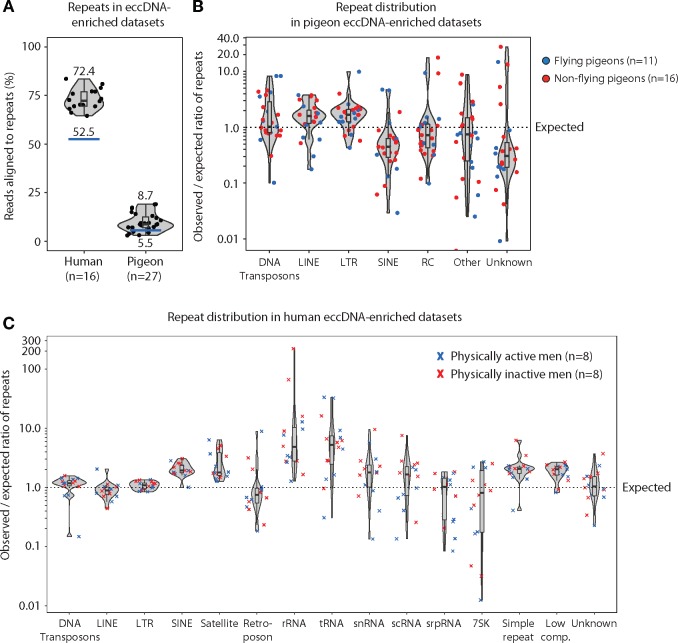
—Repetitive elements in eccDNA-enriched data sets from human and pigeon muscles. (*A*) Violin plot of observed percent repeats in human (median 72.4, *n* = 16) and pigeon (median 8.7, *n* = 27) eccDNA-enriched data sets relative to expected repeat percentage of the genome (blue line). (*B*) Violin plots of pigeon eccDNA data sets (*n* = 27), displaying observed per expected ratio of different repeat-class sequences in flying (blue) and nonflying (red) pigeons. (*C*) Violin plots of human eccDNA data sets in physically active middle-aged men (blue) (*n* = 8) and in physically inactive middle-aged men (red) corresponding to the two groups of birds, flying and nonflying, in (*B*). DNA transposon, DNA; long interspersed nuclear element, LINE; long terminal repeat, LTR; short interspersed nuclear element, SINE; rolling-circle helitron, RC; and different ribonucleic acid (RNA) repeats classes from ribosomal (rRNA), transport (tRNA), small nuclear (snRNA), small conditional (scRNA), signal recognition particle (srpRNA), and other or unknown categorized repeat features.

The pigeon genome at 1.11 Gb is ∼3-fold smaller than the 3.23-Gb human genome (Zhang, Li C, et al. 2014) and only 5.5% of the pigeon genome consist of repetitive DNA compared with 52.5% of the human genome (source: human hg38 – Dec 2013 -RepeatMasker library 20140131). To gain more insight into the consequences of repetitive DNA relative to eccDNA frequencies, we extracted eccDNA sequences from repetitive DNA of pigeon breast muscle and compared it to eccDNA from human skeletal muscle, *vastus lateralis*, purified with identical methods (Møller, Mohiyuddin, et al. 2018). The percentage of eccDNA reads that mapped to repetitive regions in the pigeon genome was 8.7% compared with an expected 5.5% of the reference genome. Similarly, eccDNA reads mapping to repetitive regions in the human genome was 72.4% compared with an expected random distribution at 52.5% (fig. 3*a*).

When looking at all obtained sequences from specific repeat classes in the eccDNA-enriched data sets, it became clear that elements encoding LINEs and long terminal repeats (LTRs) in most pigeon muscle tissues were overrepresented (1.6- and 1.7-fold, respectively) and this was regardless of breed or flight pattern (fig. 3*b*). In humans, a similar pattern was observed for SINEs (1.9-fold, median), simple repeats (2.1-fold, median), low complexity repeats (2.0-fold, median) and satellites (1.8-fold, median). These repeat elements were overrepresented in humans, whereas other repeat types were close to the expected value (∼1.0). In addition, in humans, the rRNA and tRNA gene sequences were main outliers, with larger deviation, being 4.9- and 5.3-fold more abundant than expected (fig. 3*c*). Due to the incomplete assembly of the rRNA and tRNA gene sequences in the pigeon reference genome, we were unfortunately not able to assess these repeats in the pigeon eccDNA data sets. In human to pigeon comparison, LINE and LTR’s on eccDNAs were significantly enriched in pigeons (*P* = 0.001 and *P* = 0.002, respectively), whereas SINE on eccDNAs was significantly enriched in humans (*P* < 0.0001).

In sum, our data reveal that eccDNA sequences from repetitive elements exist in proportions similar to the genome content in both the condensed genome of pigeon and the repeat-rich human genome.

### Impact of Breed, Flight, and Age on eccDNA

**Fig. 4.**
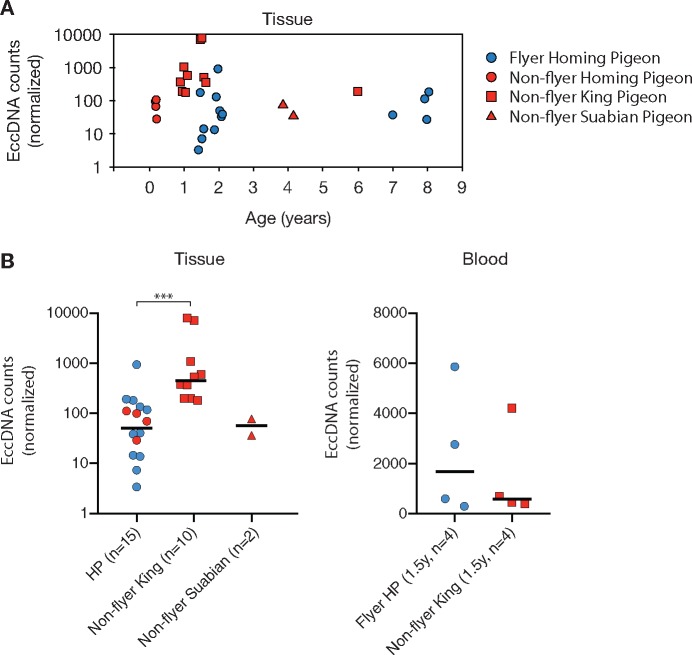
—EccDNA counts according to age, breed, and flight activity. (*A*) Detected eccDNA counts (normalized to spike-in plasmids) according to pigeon age. (*B*) Normalized eccDNA counts in pigeons from different types of breeds and with different flying behavior. Flying pigeons had flown at least 1,000–1,500 km/year. HP, homing pigeons. ***P < 0.001 Mann–Whitney test.

We next investigated if physiological factors had an impact on the number of different eccDNA in pigeon. Studies of budding yeast have revealed that ageing is associated with accumulation of rDNA-derived eccDNA (Sinclair and Guarente 1997; Shcheprova et al. 2008; Denoth Lippuner et al. 2014). To explore if ageing had impact on the frequency of eccDNA, we compared the total eccDNA count in tissue samples from pigeons with varying age (fig. 4*a* and supplementary table S2, Supplementary Material online). No linear correlation was found between age and total eccDNA counts (*n* = 27). Instead, we find that the median eccDNA count in muscle of homing pigeons remained rather constant through age: 84 eccDNA/million nuclei in 6-week-old bird (*n* = 4), 37 in 1–2-year-old birds (*n* = 8), and 78 in 7–8-year-old bird (*n* = 3) (fig. 4*a*). In king pigeons, the median number of eccDNA/million nuclei fluctuated more with age: 378 eccDNA/million in 1-year-old birds (*n* = 5), 3,845 eccDNAs in 1.5-year-old birds (*n* = 4), and 199 eccDNAs in a 6-year-old bird (*n* = 1). In a third breed, 57 eccDNAs were detected in 4-year-old Suabian pigeons (*n* = 2) (fig. 4*a*). Taken together, the data suggest that the number of unique eccDNAs does not increase with age.

Flight is associated with increased metabolic rate, oxygen consumption, and production of reactive oxidative species (Tucker 1975; Ward et al. 2002) and high metabolic activity has been suggested to elevate the risk of DNA damage (Galtier et al. 2009). To investigate if powered flight affected eccDNA frequencies, we compared the normalized eccDNA counts of the two pigeon groups, flyers and nonflyers. Flyers consisted of homing pigeons (*n* = 11) that had flown as racing pigeons (1,000–1,500 km/year) and nonflyers, consisted of preflying homing pigeons (*n* = 4, caged), caged king pigeons (*n* = 10), and caged Danish Suabian pigeons (*n* = 2). Flying and nonflying animals had anatomical features that supported a different physiology in the two groups (supplementary fig. S5, Supplementary Material online). As expected, the heart per body weight (Wright et al. 2014) was significantly higher in flying homing pigeons (*P* < 0.0001, two-tailed Mann–Whitney *U* test; supplementary fig. S5*c* and table S1, Supplementary Material online). Normalized eccDNA counts between flying (median 38, *n* = 11) and nonflying (median 84 [99, 69, 111, 29], *n* = 4) homing pigeons were similar (*P* = 0.66, two-tailed Mann–Whitney test; post hoc power = 0.025). Comparison to nonflying king pigeons revealed a significantly higher level of eccDNA, 9-fold (median 453, *n* = 10) relative to homing pigeons (median 51, *n* = 15) (fig. 4*b*, ***P = 0.0003, two-tailed Mann–Whitney test; post hoc power = 0.94). EccDNA levels in nonflying Suabian pigeons (median 57, *n* = 2) were similar to flying homing pigeons (*P* = 0.92, two-tailed Mann–Whitney test; post hoc power = 0.001) but different to king pigeons (*P* = 0.03, two-tailed Mann–Whitney test; post hoc power = 0.57). No differences were observed in blood eccDNA (fig. 4*c*, *n* = 8, *P* = 0.89, two-tailed Mann–Whitney test; post hoc power = 0.039). Hence, flight did not appear to be associated with increased eccDNA frequencies. Instead, the data suggest that eccDNA levels in muscle are affected by variation between breeds because king pigeons had significantly higher eccDNA counts than homing pigeons. Notably, post hoc power evaluation revealed that several tests had minimal statistical power (<0.9) to capture true effects. Thus, the significance on reported medians and differences remain to be assessed for these groups.

### Recurrent Genic Elements on eccDNA

**Fig. 5.**
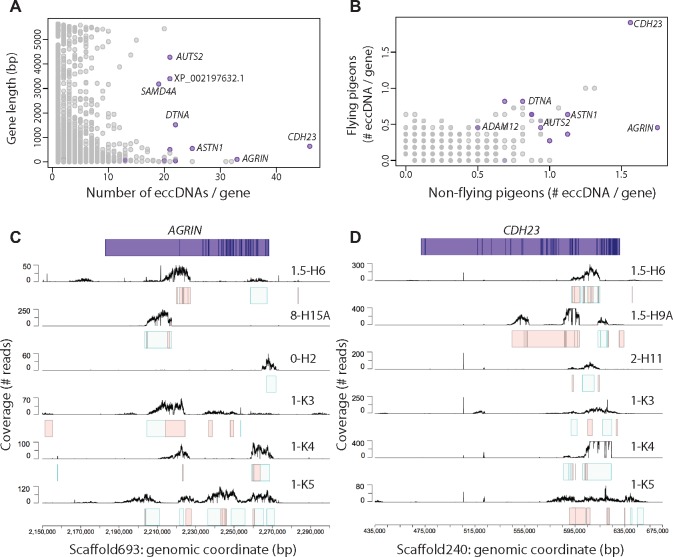
—Co-occurrence of gene-overlapping eccDNAs. (*A*) Plot of eccDNAs per gene relative to gene length, showing no apparent correlation. (*B*) Comparison of the average eccDNA count per gene in flying (*x* axis, *n* = 11) and nonflying (*y* axis, *n* = 16) pigeons. (*C*, *D*) Schematic representation of the two most eccDNA-rich genes *AGRIN* and *CDH23* with exons displayed as dark-blue lines. Mapping profiles of eccDNA from *AGRIN* and *CDH23* in six animals (black lines). Intervals for the corresponding eccDNAs are displayed as light green boxes, whereas light red boxes designate additional putative eccDNA mapping profiles that were below threshold cutoff for detection of eccDNA.

Next, we assessed the frequency of eccDNAs in annotated genes and their co-occurrence among different pigeons (fig. 5*a*). We found that eccDNAs were enriched for a subset of pigeon genes and present in several birds (fig. 5*a* and supplementary table S4, Supplementary Material online). Enrichment was independent of gene length (fig. 5*a*) and genes were found both in flying and nonflying pigeons (fig. 5*b*). In addition, certain eccDNAs derived repeatedly from the same genes but from different positions within the genes (e.g., *AGRIN*, fig. 5*c*). Other types of frequently detected eccDNAs had breakpoints in or in close proximity to genes and overlapped at high percentage in bird-to-bird comparison (e.g., *CDH23*, fig. 5*d*).

Some of the recurrent genes on eccDNAs were related to energy metabolism, muscle formation, and maintenance (e.g., *AGRIN*, *DTNA*, and *ADAM12*, fig. 5*a*–*c*). The *AGRIN* gene, found to be enriched in nonflying pigeons (fig. 5*b* and *c*), is particularly interesting. The human ortholog encodes a large cell surface protein involved in the development of the neuromuscular junction. If impaired or mutated, it can lead to muscle weakness and decremental muscle response in humans (known as congenital myasthenic syndrome) (Nicole et al. 2014). The *DTNA* gene, found on 22 eccDNAs and equally present among different pigeon breeds (fig. 5*a* and *b*), encodes dystrobrevin, which is involved in connecting the cytoskeleton of a muscle fiber to the surrounding extracellular matrix through the cell membrane (Grady et al. 2003). The gene *CDH23*, encoding cadherin related 23, gave rise to most eccDNAs per gene in both pigeon groups (fig. 5*b* and *d*). The human *CDH23* ortholog is required for normal hearing (*CDH23* mutations lead to deafness, Usher syndrome [Bork et al. 2001; Astuto et al. 2002]) but so far *CDH23* and mammalian orthologs have not been linked to muscle function. Overall, these results suggest that a number of genes could be prone to be captured more frequently on eccDNAs than others.

**Fig. 6.**
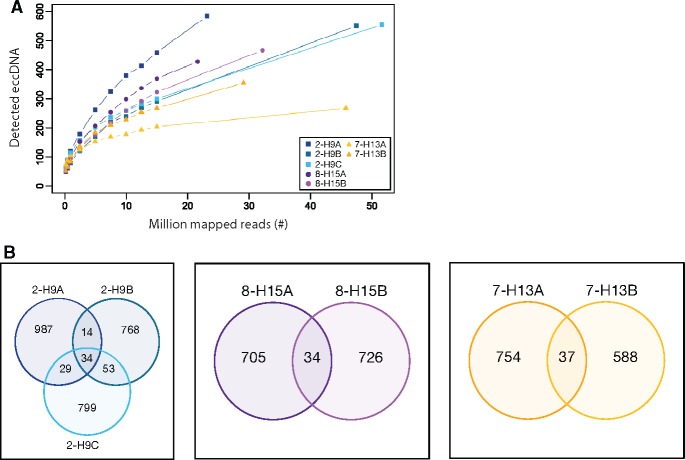
—EccDNA in biological replicates from muscle. (*A*) Decimation plot showing million mapped reads relative to detected eccDNA counts from replicates of three different homing pigeons 2-H9, 8-H15, and 7-H13 (*n* = 3, *n* = 2, and *n* = 2). (*B*) Venn diagrams of eccDNA recurring in biological replicates from homing pigeons 2-H9, 8-H15, and 7-H13.

We furthermore explored the impact of sequencing depth relative to eccDNA detection and found similar trends between samples (supplementary figs. S6 and S7, Supplementary Material online). The sequencing of 34–66 million reads per sample (median 51.9 million) suggested that most eccDNAs were detected, as trajectories for the majority of samples were toward saturation plateaus. However, it also implied that additional sequencing could have captured more eccDNAs, especially for blood samples (supplementary fig. S7, Supplementary Material online). To understand if this had a significant impact on quantification of eccDNA, we accessed the variation within an animal. The number of detected eccDNAs within the same pigeon (breast tissue) revealed substantially less SD between eccDNA counts: 915, 869, and 1,064 ± 102 SD (*n* = 3); 760 and 739 ± 15 SD (*n* = 2); 791 and 625 ± 117 SD (*n* = 2) (fig. 6*a*) compared with the biological variation between different homing pigeons: 739 ± 696 SD (*n* = 15) or king pigeons: 1,158 ± 1,908 SD (*n* = 10) (supplementary fig. S6, Supplementary Material online). This implied that differences in eccDNA counts between animals mainly represented biological variation. Interestingly, the chromosomal origin of eccDNAs among technical replicates differed substantially (overlap between 3.5% and 4.6%) suggesting that, although eccDNA counts were similar, the eccDNAs were likely formed independently in adjacent muscle compartments (fig. 6*b*). An observation also found in biological replicates of human leukocytes (Møller, Mohiyuddin, et al. 2018).

## Discussion

Here, we describe genome-wide mapping of eccDNAs from a vertebrate genome that has condensed through evolution and contains a lower proportion of noncoding and repetitive DNA compared with mammalian genomes. We find that eccDNAs in pigeons form from all types of elements, including genic, intergenic, and repetitive regions (fig. 2). Some early eccDNA studies have focused on circular DNA with repetitive elements (Rush and Misra 1985; Gaubatz 1990; Tomaska et al. 2004; Cohen and Segal 2009 for reviews), whereas others have identified eccDNA from unique regions of vertebrate genomes (Bertelsen et al. 1982; Stanfield and Helinski 1984, 1986; van Loon et al. 1994). Our data reveal that repetitive elements on eccDNAs exist in proportions similar to the distribution in the pigeon genome, as eccDNAs from exons, genes, and LINEs or LTRs only were overrepresented by maximal 1.7-fold (figs. 2 and 3*b*). Thus, our data support that both unique and repetitive genomic sequences are equal sources of eccDNA.

Half of the human genome consists of repetitive DNA such as DNA transposons, satellites, and simple repeats, whereas the pigeon genome only contains 5.5% repeats (fig. 3*a*). One expectation could be that due to this large difference in repetitive content DNA circularization frequencies would be affected at sites of DNA breakages, possibly as the chance of ectopic intrachromatid recombination could be higher in human DNA relative to pigeon DNA. However, we find that this is generally not the case for the majority of repeat classes (fig. 3*b* and *c*). Sequences from most repetitive elements in eccDNA were generally present in the same or in lower proportions compared with the genomic reference of pigeon and human. In the pigeon genome, SINEs are rare and mostly inactive (0.09% of *C. livia* genome [Zhang, Li C, et al. 2014]) and this could explain why SINE sequences were less represented than expected by chance, in contrast to LINEs (4.2% of *C. livia* genome [Zhang, Li C, et al. 2014]) (fig. 3*b*). In human genomes, SINEs, satellites (1.9- and 1.8-fold, median), and in particular sequences of rRNA and tRNA genes (4.9- and 5.3-fold, median) were enriched (fig. 3*c*). The ∼2-fold higher abundance of SINEs (13.4% of the human genome) on eccDNAs in humans may be associated with the retrotransposition process (Singer 1982; Jones and Potter 1985) as well as their prevalence in the genome. Clustering of satellites in tandem-repeats (2.6% of the human genome) may explain the high satellite-eccDNA frequency because short genetic distance is known to increase rates of deletions (Campbell and Eichler 2013), which can give rise to eccDNA (Gresham et al. 2010; Møller, Lin, et al. 2018). The overrepresentation of tRNA genes in human eccDNA might also be caused by clustering of tRNA genes in the genomes. There are 277 individual tRNA clusters scattered across the genome with up to 29 tRNA genes per cluster (Van Bortle et al. 2017). Likewise, more than thousand ribosomal RNA genes (1,317 5S rRNA, 454 LSU-rRNA, and 90 SSU-rRNA) are found in the human genome with nearly half of them clustered on chromosome 1. The rest are clustered on p-arms of chromosome 13, 14, 15, 21, and 22, which all could be hot spots for eccDNA formation as seen in other eukaryotes (Cohen 2003; Mansisidor et al. 2018). Segal and coworkers previously revealed that human eccDNA emerge from 5S rRNA repeats as well as from satellites (Cohen et al. 2010), consistent with recent findings (Møller, Mohiyuddin, et al. 2018). In addition, a higher and/or coordinated transcription of 5S rRNA could potentially also result in a higher eccDNA frequency, as high transcription rate has been shown to increase mutation rates/copy number variation in some eukaryotic cells (Thomas and Rothstein 1989; Hull et al. 2019). In pigeon and human comparison of eccDNA counts, we detected ∼9–10 times more eccDNAs per human nucleus, which is 3 times above the expectation if one assumes that eccDNA frequencies correlate with genome length (∼3 times larger human genome). Yet, more studies are needed to deduce what type of potential connection there is between genome length and eccDNA frequencies.

The current study reveals that a small subset of pigeon genes is also enriched on eccDNA. We did not find a correlation to structural or functional features that could explain why some genes were more prone to form eccDNA than others, except for agrin and the dystrobrevin genes, *AGRIN* and *DNTA1*, that are essential for muscle function. This could suggest that genes expressed in the muscle tissue are more prone to form eccDNA. Two independent studies of human and worm (*Caenorhabditiselegans*) muscle tissue have shown that the most highly expressed gene in muscle, *TTN*, forms the most eccDNA (Shoura et al. 2017; Møller, Mohiyuddin, et al. 2018). However, no general correlation has been found between transcription and the number of eccDNAs formed from genes. Therefore, the high propensity for certain genes to form eccDNA still enquires further investigation to unravel the underlying mechanism.

We find significantly more eccDNA elements from muscle tissue in nonflying king pigeons, obtained from two different pigeon breeders (supplementary table S1, Supplementary Material online), compared with homing pigeons (fig. 4). This result suggests that the genetic load from eccDNA can change significantly between members of the same species, having practically identical genome sequences (Zhang, Li B, et al. 2014). This could have, yet uncovered, physiological consequences. For instance, the higher amount of eccDNA types in king pigeons could potentially have impact on life span as eccDNA can carry proto-oncogenes that are shown to correlate with tumorigenesis (Von Hoff et al. 1992; Nathanson et al. 2014; Turner et al. 2017; Wu et al. 2019).

The biological variation observed between eccDNA types within nuclei from a pigeons’ breast muscle tissue, just millimeters apart, might reflect that different stem cell progenitor cells gave rise to the tissue and accumulated distinct types of mutations. Moreover, as acentric eccDNAs will not segregate faithfully in mitosis, these mutations are expected to be present in a limited number of cells. The eccDNA distribution among cells would be even less pronounced for eccDNAs with no or limited DNA replication. Still, we cannot completely exclude that the low overlap between replicative samples is an artifact, though we attempted to avoid this by generally performing eccDNA purification and amplification of samples in a scrambled order, using excess amounts of random hexamer oligos and phi29 polymerase to unbiasedly amplify eccDNAs and to detect these after extensive sequencing.

Besides flight and breed, we also looked at the influence of age on eccDNA frequencies. Previous studies of yeast have shown that eccDNA from rDNA (ERC) accumulates in aging cells and accumulation correlates with cellular senescence and a shorter life span of the cell (Sinclair and Guarente 1997; Shcheprova et al. 2008; Denoth Lippuner et al. 2014). On the other hand, brain, liver, and heart cells in mice were shown not to accumulate Alu-elements (SINEs) on eccDNA as a function of age but rather declined (Gaubatz and Flores 1990). Based on data-points from 15 homing pigeons and 10 king pigeons, a potential age correlation could not be established (fig. 4*a*). Thus, the current study does not support any correlation between age and eccDNA frequency.

Finally, this study supports that eccDNA is a common element in pigeons. It supplements previous genome-scale eccDNA results from *Caenorhabditis elegans* (worm), *Saccharomyces cerevisiae* (yeast), *Oxytricha* (ciliate), mouse, and human tissue, cell lines, and plasma (Shibata et al. 2012; Møller, Parsons, et al. 2015; Møller et al. 2016; Kumar et al. 2017; Shoura et al. 2017; Zhu et al. 2017; Yerlici et al. 2019) by showing that the majority of eccDNAs from somatic tissue are smaller than 1 kb and that a fraction of eccDNAs include exons and genic sequences, which could influence cellular processes in eukaryotic cells.

## Supplementary Material

Supplementary data are available at *Genome Biology and Evolution* online.

## Supplementary Material

evz281_Supplementary_Data

## Notes

**Data deposition:** This project has been deposited at European Nucleotide Archive under the accession PRJEB34837.
