# Supplementary material for: Near-Random Distribution of Chromosome-Derived Circular DNA in the Condensed Genome of Pigeons and the Larger, More Repeat-Rich Human Genome
Source: Genome Biol Evol. 2019 Dec 27;12(2):3762–77. doi: 10.1093/gbe/evz281 (PMC6993614; doi:10.1093/gbe/evz281)
Supplement: evz281_Supplementary_Data [file evz281_supplementary_data.zip › Supplementary figure and table legends.docx]

**Supplementary figure and table legends**

**Fig. S1. Genomic input material from pigeon tissue and blood.** A) Amount of dried breast muscle fibers in mg from 32 samples used as input material for Circle-Seq and genomic DNA purification. B) Genomes per 2 mg muscle lysate estimated from total nanogram (ng) purified genomic DNA. C-D) Quantification of genomic input from 2 mg muscle estimated by *EFBN2* gene copies, showing (C) quantitative PCR (qPCR) CT values and (D) calculated *EFBN2* copies per sample. E) Genomes per blood sample estimated from ng DNA. F-G), As in C and D panels, quantifying genomic input from blood. Flyers, blue; non-flyers, red; median value, dotted line. Representative qPCR data shown (C-D, F-G). Each qPCR reaction was performed in quadruplicates and performed three independent times.

**Fig. S2. Quantified DNA reminiscence after exonuclease.** Quantification of remaining DNA after 5-day exonuclease treatment of A-B) tissue and C-D) blood, showing CT values (A+C) and *EFBN2* copies per sample (B+D). Flyers, blue; non-flyers, red; median value, dotted line; standard deviation, black line. Representative qPCR data are shown. Each qPCR reaction was performed in quadruplicates and performed minimum two times.

**Fig. S3.** **Plasmid and mitochondrial DNA read coverage**. A) Percent reads mapped to spike-in plasmid controls and B) mitochondrial DNA.

**Fig. S4. Blastn and taxonomy assignment of 10,000 random selected reads.** A) Blastn analysis reveals abundant viral DNA (circovirus) relative to aves, bacteria and other species in homing pigeon 8-H14. B-C) As A, displaying distribution in breast muscle tissue from B) homing pigeons and C) king pigeons. D) Distribution in blood from flying and non-flying pigeons. B-D) Bacterial DNA, primarily consisting of benign, gram-positive *Rhodococcus erythropolis.*

**Fig. S5. Weight, wingspan and anatomical ratio per body weight of pigeons.**

A) Measured weight in grams of body, entire breast muscle (right-side) and heart of homing pigeons (HP) and king pigeons. B) Measured wingspans in centimeters of each bird. C-E) Separated by pigeon type, plots shows the anatomical ratio of: C) heart weight (grams) per bird body weight (grams); D) breast muscle weight (grams) per bird body weight (grams); E) length of wingspan (cm) per bird body weight (grams).

**Fig. S6. Effect of sequencing level on eccDNA detection in muscle**. EccDNA counts as a function of mapped reads (randomly sampled), displayed as decimation plots for muscle tissue: A) non-flying king and Suabian pigeons, B) non-flying and flying homing pigeons.

**Fig. S7. Effect of sequencing level on eccDNA detection in blood**. EccDNA counts as a function of mapped reads (randomly sampled), displayed as decimation plots for blood from 1.5-year-old pigeons: A) counted eccDNAs with high confidence (hconf), B) total counted eccDNAs including eccDNA with low confidence.

**Table S1. Physiological data on collected pigeons.** Table with 12 columns, from left: ID, pigeon short identification name; Old ID, original pigeon name (ring sign); Pigeon type, breed; Sample type, breast muscle tissue or blood; Sex, M (male), F (female), ? (not known); Age, approximately age in years; Body weight, animal in gram; Wing-span, stretched maximum in centimeters; Breast muscle, weight of the entire right-side breast muscle in gram; Heart, weight in gram; Heart/Body weight, measured in grams; Source, location of domestic pigeon breeder.

**Table S2. Detected eccDNA counts and bioinformatics flag stats.**

**Table S3. Overrepresentation of genomic features on eccDNA relative to expected by chance.** Table with 7 columns, from left: genomic feature; eccDNA size category; detected, number of eccDNAs observed among all samples; expected, number of eccDNAs observed in a randomization of the observed eccDNA positions; total eccDNAs, sum of eccDNA per category; p-value, calculated by chi-square test; p-value summary, p < 0.05 (*); p < 0.01 (**); p < 0.001 (***); p < 0.0001 (****).

**Table S4. Total eccDNA counts relative to random counts at genomic features.** *Expected represent average after simulating overlaps of ten independent sets (drawn from a similar length distribution as eccDNAs) of randomly selected regions and their corresponding genomic features.
