# Supplementary material for: Near-Random Distribution of Chromosome-Derived Circular DNA in the Condensed Genome of Pigeons and the Larger, More Repeat-Rich Human Genome
Source: Genome Biol Evol. 2019 Dec 27;12(2):3762–77. doi: 10.1093/gbe/evz281 (PMC6993614; doi:10.1093/gbe/evz281)
Supplement: evz281_Supplementary_Data [file evz281_supplementary_data.zip › Table S1.pdf]

Table S1

| ID | Old ID | Pigeon type | Total flight in competition (km) | Sample type | SEX | AGE (years) | Body weight (g) | Wing-span (cm) | Breast muscle (g) | Heart (g) | Heart (g) per Body weight (g) | Domestic pigeon breeder |
|---|---|---|---|---|---|---|---|---|---|---|---|---|
| **0_H1** | HP1 | Homing non-flyer | - | tissue | ? | 0.1 | 378 | 56 | 37.44 | 5.85 | 0.0155 | F. Nygaard, Vordingborg, Denmark |
| **0_H2** | HP4.2 | Homing non-flyer | - | tissue | ? | 0.1 | 360 | 56.5 | 38.841 | 5.703 | 0.0158 | F. Nygaard, Vordingborg, Denmark |
| **0_H3** | HP6 | Homing non-flyer | - | tissue | ? | 0.1 | 425 | 63 | 41.064 | 5.565 | 0.0131 | F. Nygaard, Vordingborg, Denmark |
| **0_H4** | HP7.1 | Homing non-flyer | - | tissue | ? | 0.1 | 361 | 55 | 29.789 | 4.735 | 0.0131 | F. Nygaard, Vordingborg, Denmark |
| **1.5_H5** | HP304 | Homing flyer | 1573 | tissue | M | 1.5 | 362 | 66 | 49.39 | 6.78 | 0.0187 | F. Nygaard, Vordingborg, Denmark |
| **1.5_H6** | HP307 | Homing flyer | 1717 | tissue | F | 1.5 | 391 | 68 | 46.53 | 6.83 | 0.0175 | F. Nygaard, Vordingborg, Denmark |
| **1.5_H7** | HP354 | Homing flyer | 1064 | tissue | M | 1.5 | 431 | 67 | 53.07 | 7.19 | 0.0167 | F. Nygaard, Vordingborg, Denmark |
| **1.5_H8** | HP361 | Homing flyer | 1883 | tissue | F | 1.5 | 363 | 65 | 41.71 | 6.24 | 0.0172 | F. Nygaard, Vordingborg, Denmark |
| **2_H9A** | HP777A | Homing flyer | 1867 | tissue | M | 2 | 635 | 69 | 72.09 | 8.543 | 0.0135 | F. Nygaard, Vordingborg, Denmark |
| **2_H9B** | HP777B | Homing flyer | 1867 | tissue | M | 2 | 635 | 69 | 72.09 | 8.543 | 0.0135 | F. Nygaard, Vordingborg, Denmark |
| **2_H9C** | HP777C | Homing flyer | 1867 | tissue | M | 2 | 635 | 69 | 72.09 | 8.543 | 0.0135 | F. Nygaard, Vordingborg, Denmark |
| **2_H10** | HP784 | Homing flyer | 2305 | tissue | F | 2 | 586 | 67 | 67.87 | 7.57 | 0.0129 | F. Nygaard, Vordingborg, Denmark |
| **2_H11** | HP801 | Homing flyer | 2058 | tissue | M | 2 | 575 | 69 | 64.63 | 7.3 | 0.0127 | F. Nygaard, Vordingborg, Denmark |
| **2_H12** | HP981 | Homing flyer | 733 | tissue | F | 2 | 554 | 59 | 60.96 | 6.5 | 0.0117 | F. Nygaard, Vordingborg, Denmark |
| **7_H13B** | HP082B | Homing flyer | NA | tissue | F | 7 | NA | NA | NA | NA | NA | G. Sørensen, Næstved, Denmark |
| **7_H13A** | HP082A | Homing flyer | NA | tissue | F | 7 | NA | NA | NA | NA | NA | G. Sørensen, Næstved, Denmark |
| **8_H14** | HP286 | Homing flyer | 4217 | tissue | M | 8 | 579 | 70 | 57.24 | 6.72 | 0.0116 | F. Nygaard, Vordingborg, Denmark |
| **8_H15A** | HP415A | Homing flyer | 5243 | tissue | F | 8 | 637 | 59 | 68.82 | 7.19 | 0.0113 | F. Nygaard, Vordingborg, Denmark |
| **8_H15B** | HP415B | Homing flyer | 5243 | tissue | F | 8 | 637 | 59 | 68.82 | 7.19 | 0.0113 | F. Nygaard, Vordingborg, Denmark |
| **1_K1** | K404 | King non-flyer | - | tissue | M | 1 | 653 | 73 | 68 | 5.3 | 0.0081 | H. Pedersen, Holstebro, Denmark |
| **1_K2** | K433 | King non-flyer | - | tissue | M | 1 | 715 | 72 | 73 | 6.9 | 0.0097 | H. Pedersen, Holstebro, Denmark |
| **1_K3** | K438 | King non-flyer | - | tissue | M | 1 | 789 | 72 | 64 | 7.46 | 0.0095 | H. Pedersen, Holstebro, Denmark |
| **1_K4** | K440 | King non-flyer | - | tissue | F | 1 | 703 | 69 | 69 | 6.09 | 0.0087 | H. Pedersen, Holstebro, Denmark |
| **1_K5** | K446 | King non-flyer | - | tissue | M | 1 | 733 | 69 | 65 | 6.46 | 0.0088 | H. Pedersen, Holstebro, Denmark |
| **1.5_K6** | K1569 | King non-flyer | - | tissue | F | 1.5 | 621 | 72 | 54.51 | 6.82 | 0.0110 | H. Christiansen, Zealand, Denmark |
| **1.5_K7** | K1570 | King non-flyer | - | tissue | M | 1.5 | 643 | 71 | 65.35 | 5.41 | 0.0084 | H. Christiansen, Zealand, Denmark |
| **1.5_K8** | K1697 | King non-flyer | - | tissue | M | 1.5 | 488 | 72 | 39.95 | 5.49 | 0.0113 | H. Christiansen, Zealand, Denmark |
| **1.5_K9** | K2765 | King non-flyer | - | tissue | M | 1.5 | 608 | 68 | 55.95 | 6.48 | 0.0107 | H. Christiansen, Zealand, Denmark |
| **6_K10** | K210 | King non-flyer | - | tissue | M | 6 | 755 | 65 | 89 | 6.72 | 0.0089 | H. Pedersen, Holstebro, Denmark |
| **4_S1** | S100 | Suabian non-flyer | - | tissue | M | 4 | NA | NA | NA | NA | NA | A. Christensen, Næstved, Denmark |
| **4_S2** | S101 | Suabian non-flyer | - | tissue | F | 4 | NA | NA | NA | NA | NA | A. Christensen, Næstved, Denmark |
| **B1.5_H5** | HP304 | Homing flyer | 1573 | blood | M | 1.5 | 362 | 66 | 49.39 | 6.78 | 0.0187 | F. Nygaard, Vordingborg, Denmark |
| **B1.5_H6** | HP307 | Homing flyer | 1717 | blood | F | 1.5 | 391 | 68 | 46.53 | 6.83 | 0.0175 | F. Nygaard, Vordingborg, Denmark |
| **B1.5_H7** | HP354 | Homing flyer | 1064 | blood | M | 1.5 | 431 | 67 | 53.07 | 7.19 | 0.0167 | F. Nygaard, Vordingborg, Denmark |
| **B1.5_H8** | HP361 | Homing flyer | 1883 | blood | F | 1.5 | 363 | 65 | 41.71 | 6.24 | 0.0172 | F. Nygaard, Vordingborg, Denmark |
| **B1.5_K6** | K1569 | King non-flyer | - | blood | F | 1.5 | 621 | 72 | 54.51 | 6.82 | 0.0110 | H. Christiansen, Zealand, Denmark |
| **B1.5_K7** | K1570 | King non-flyer | - | blood | M | 1.5 | 643 | 71 | 65.35 | 5.41 | 0.0084 | H. Christiansen, Zealand, Denmark |
| **B1.5_K8** | K1697 | King non-flyer | - | blood | M | 1.5 | 488 | 72 | 39.95 | 5.49 | 0.0113 | H. Christiansen, Zealand, Denmark |
| **B1.5_K9** | K2765 | King non-flyer | - | blood | M | 1.5 | 608 | 68 | 55.95 | 6.48 | 0.0107 | H. Christiansen, Zealand, Denmark |
