# Supplementary material for: Near-Random Distribution of Chromosome-Derived Circular DNA in the Condensed Genome of Pigeons and the Larger, More Repeat-Rich Human Genome
Source: Genome Biol Evol. 2019 Dec 27;12(2):3762–77. doi: 10.1093/gbe/evz281 (PMC6993614; doi:10.1093/gbe/evz281)
Supplement: evz281_Supplementary_Data [file evz281_supplementary_data.zip › Table S2.pdf]

Table S2

ID	Old ID	Pigeon Type	Sample origin	Total sequenced reads	Paired-end reads	Total mapped reads*	Total mapped to nuclear genome	Total mapped mtDNA reads	Total mapped reads to plasmids	Putative detected eccDNA (incl. low quality)	Detected eccDNA	Reads mapped to detected eccDNA	Reads mapped to detected eccDNA	EccDNA (normalized to total plasmid reads in %)	% mapped reads to total plasmids out of TOTAL mapped reads
**0_H1**	HP1	Homing non-flyer	breast muscle	52762712	52665546	32149536	29610252	55919	2483365	2058	761	27082660	20415602	98.5	7.724
**0_H2**	HP4.2	Homing non-flyer	breast muscle	48792366	48657806	26265333	21965395	41726	4258212	3438	1124	22550155	15038201	69.3	16.212
**0_H3**	HP6	Homing non-flyer	breast muscle	54922266	54785722	28942552	25290063	66735	3585754	3431	1372	23629608	17677278	110.7	12.389
**0_H4**	HP7	Homing non-flyer	breast muscle	51494744	51232754	17570920	14581882	8166	2980872	1082	490	14929704	7582739	28.9	16.965
**1.5_H5**	HP304	Homing flyer	breast muscle	55212896	53428162	1176374	1092663	56	83655	84	24	661516	494098	3.4	7.111
**1.5_H6**	HP307	Homing flyer	breast muscle	56666564	56560690	42426905	36137070	2379983	3909852	3828	1671	34918539	28768527	181.3	9.216
**1.5_H7**	HP354	Homing flyer	breast muscle	64615854	64331182	1514293	1389855	123	124315	154	60	755825	703295	7.3	8.209
**1.5_H8**	HP361	Homing flyer	breast muscle	34263346	32357080	23479775	17438455	10273	6031047	694	374	17528620	13975263	14.6	25.686
**2_H10**	HP784	Homing flyer	breast muscle	44474832	44395000	20860836	13322865	20886	7517085	1223	494	16391679	13339141	13.7	36.034
**2_H11**	HP801	Homing flyer	breast muscle	46845066	46642620	29663059	26538019	109391	3015649	3814	1365	23634019	17475924	134.3	10.166
**2_H12**	HP981	Homing flyer	breast muscle	51228030	51120220	44657304	43358387	42692	1256225	9418	2642	36219699	22056493	939.2	2.813
**2_H9A**	HP777(A)	Homing flyer	breast muscle	46002474	45902862	23122385	18260978	76355	4785052	2583	1064	17590545	12694074	51.4	20.694
**2_H9B**	HP777(B)	Homing flyer	breast muscle	58008300	57783056	47408316	35223022	31448	12153846	2163	869	32483280	27966999	33.9	25.637
**2_H9C**	HP777(C)	Homing flyer	breast muscle	56019230	55810346	51544491	39857485	12828	11674178	2091	915	36988439	27881592	40.4	22.649
**7_H13A**	HP082(A)	Homing flyer	breast muscle	47507628	47401038	21621339	17341442	124279	4155618	1861	739	17253326	14764078	38.4	19.220
**7_H13B**	HP082(B)	Homing flyer	breast muscle	51934938	51773972	48306667	44049596	4245648	11423	2094	760	43118478	20237710	32139.6	0.024
**8_H14**	HP286	Homing flyer	breast muscle	51892092	51242598	14505221	13966474	253655	285092	384	231	13227147	12932837	117.5	1.965
**8_H15A**	HP415(A)	Homing flyer	breast muscle	51456798	51358598	29076123	22530626	46854	6498643	1403	625	18237644	13051047	28.0	22.350
**8_H15B**	HP415(B)	Homing flyer	breast muscle	51469218	51273722	45691176	43152683	642532	1895961	1780	791	39960062	14706970	190.6	4.150
**B1.5_H5**	HPB304	Homing flyer	blood	56172882	55967186	51369699	51090693	595	278411	6378	1497	35782154	24020758	2762.1	0.542
**B1.5_H6**	HPB307	Homing flyer	blood	50729230	50461432	39387866	38239210	311	1148345	2933	873	30460063	21345468	299.4	2.915
**B1.5_H7**	HPB354	Homing flyer	blood	59663448	59377398	50158654	49270775	333	887546	3848	1051	37662525	25718091	594.0	1.769
**B1.5_H8**	HPB361	Homing flyer	blood	54507612	54296442	47944826	47807324	803	136699	7658	1671	32284884	18502664	5860.7	0.285
**1_K1**	KP404	King non-flyer	breast muscle	49648654	49439078	41670242	40661441	232187	776614	1681	705	32361463	22567132	378.3	1.864
**1_K2**	KP433	King non-flyer	breast muscle	54765884	54525248	30509641	28895831	85381	1528429	3035	994	22593122	13148449	198.4	5.010
**1_K3**	KP438	King non-flyer	breast muscle	57487178	57279188	45176004	44234336	422364	519304	3950	1239	34332634	25268112	1077.8	1.150
**1_K4**	KP440	King non-flyer	breast muscle	50386604	50073690	22534029	21668609	31188	834232	1955	672	15019483	9026205	181.5	3.702
**1_K5**	KP446	King non-flyer	breast muscle	52492608	52202772	42057083	41125803	179428	751852	3608	1077	31055659	19155237	602.5	1.788
**1.5_K6**	KP1569	King non-flyer	breast muscle	41353446	41265622	39804858	39364036	141644	299178	10694	5384	31962525	19748279	7163.3	0.752
**1.5_K7**	KP1570	King non-flyer	breast muscle	49374074	49286738	44549933	42258298	141842	2149793	6043	2544	36591520	16758802	527.2	4.826
**1.5_K8**	KP1697	King non-flyer	breast muscle	53244834	53160332	49624930	49084873	182970	357087	22889	5744	36253387	19470919	7982.5	0.720
**1.5_K9**	KP2765	King non-flyer	breast muscle	49545602	49457846	42183432	39715495	81645	2386292	6066	2096	33675327	17761037	370.5	5.657
**6_K10**	KP210	King non-flyer	breast muscle	51790512	51424598	27226010	25881753	150658	1193599	2796	872	20336694	11020599	198.9	4.384
**B1.5_K6**	KPB1569	King non-flyer	blood	66403766	66041734	48788982	47631838	452	1156692	3007	934	40145988	22931599	394.0	2.371
**B1.5_K7**	KPB1570	King non-flyer	blood	60372956	60132314	50279773	49713912	335	565526	2913	786	40672349	24819023	698.8	1.125
**B1.5_K8**	KPB1697	King non-flyer	blood	57186336	56939868	49411677	49317912	293	93472	3249	796	40852797	29110801	4207.9	0.189
**B1.5_K9**	KPB2765	King non-flyer	blood	57360806	57116434	45676406	44621317	335	1054754	3817	1066	35578189	22178226	461.6	2.309
**4_S1**	SB100	Suabian non-flyer	breast muscle	49556892	49362944	47410117	41594812	17974	5797331	2069	943	32879175	25976680	77.1	12.228
**4_S2**	SB101	Suabian non-flyer	breast muscle	54721692	54492664	51417551	39871281	23403	11522867	2377	803	37943878	31297651	35.8	22.410
