# Supplementary material for: Near-Random Distribution of Chromosome-Derived Circular DNA in the Condensed Genome of Pigeons and the Larger, More Repeat-Rich Human Genome
Source: Genome Biol Evol. 2019 Dec 27;12(2):3762–77. doi: 10.1093/gbe/evz281 (PMC6993614; doi:10.1093/gbe/evz281)
Supplement: evz281_Supplementary_Data [file evz281_supplementary_data.zip › Table S3.pdf]

Table S3

Genomic feature	Size category	Detected	Expected	Total eccDNA	P-value	P-value summary
Exons	all	5'176	4'184.80	29'327	6.28E-29	****
	1-1000 bp	1'055	783.20	15'963	7.45E-11	****
	1000-50000 bp	4'095	3'384.10	13'336	3.73E-22	****
	>50000	26	17.50	28	1.41E-02	*
Genes	all	9'650	9'139.30	29'327	6.39E-06	****
	1-1000 bp	4'355	4'445.90	15'963	2.60E-01	ns
	1000-50000 bp	5'269	4'675.90	13'336	6.57E-14	****
	>50000	26	17.50	28	1.41E-02	*
Repetitive elements	all	9'495	8'985.70	29'327	6.32E-06	****
	1-1000 bp	2'045	1'536.20	15'963	2.07E-19	****
	1000-50000 bp	7'422	7'421.70	13'336	1.00E+00	ns
	>50000	28	27.80	28	ND	ND
5'UTRs	all	1'897	1'301.20	29'327	2.20E-16	****
	1-1000 bp	169	103.10	15'963	7.56E-05	****
	1000-50000 bp	1'704	1'184.80	13'336	2.20E-16	****
	>50000	24	13.30	28	4.77E-03	**
LINE	all	7671	7186	29'327	4.32E-06	****
	1-1000 bp	1673	1197	15'963	1.48E-20	****
	1000-50000 bp	5970	5961	13'336	9.22E-01	ns
	>50000	28	28	28	9.22E-01	ns
DNA transposon	all	1364	1494	29'327	1.34E-02	*
	1-1000 bp	87	109	15'963	1.33E-01	ns
	1000-50000 bp	1265	1367	13'336	3.81E-02	*
	>50000	12	18	28	1.80E-01	ns
LTR	all	1980	2007	29'327	6.70E-01	ns
	1-1000 bp	255	198	15'963	8.03E-03	**
	1000-50000 bp	1712	1790	13'336	1.63E-01	ns
	>50000	13	20	28	1.03E-01	ns
SINE	all	602	567	29'327	3.15E-01	ns
	1-1000 bp	34	38	15'963	7.23E-01	ns
	1000-50000 bp	562	520	13'336	2.03E-01	ns
	>50000	6	9	28	6.01E-01	ns
RC/Helitron	all	83	85	29'327	9.39E-01	ns
	1-1000 bp	0	4	15'963	1.34E-01	ns
	1000-50000 bp	82	79	13'336	8.74E-01	ns
	>50000	1	2	28	1.00E+00	ns
Unknown	all	226	263	29'327	1.02E-01	ns
	1-1000 bp	21	17	15'963	6.26E-01	ns
	1000-50000 bp	202	239	13'336	8.39E-02	ns
	>50000	3	6	28	4.67E-01	ns
GC content (%)	all	47.448	41.560	29'327	< 2.2E-16	****
	1-1000 bp	49.633	41.570	15'963	< 2.2E-16	****
	1000-50000 bp	44.821	41.549	13'336	< 2.2E-16	****
	>50000	51.796	41.544	28	3.73E-08	****
